# Fellow-Workers and Their Doings

**Published:** 1914-08-01

**Authors:** 


### ROUND THE WORLD.

### Fellow-Workers and their Doings.

[The Editor Invites Early Information and Contributions Marked "Fellow-Workers."]

The new president of the Medical Defence Union, Sir John Tweedy, F.R.C.S., is famous as an ophthalmic surgeon who for many years served in that capacity at the hospital where he first entered as a student (University College), and is one of that small but distinguished band who have passed the presidential chair of the Royal College of Surgeons. He has always taken a prominent part in the domestic affairs of the medical profession in this country, and is at the present time president of the Royal Medical Benevolent Fund as well as a trustee of the Hunterian collection in Lincoln's Inn Fields.

Some interesting work has lately been done by Mr. A. F. Stanley Kent, M.A. Oxon., the Wills Professor of Physiology at Bristol. Taking for his quest suggested connecting bundles of fibres between the two main divisions of the heart (auricle and ventricle), Mr. Kent has succeeded in demonstrating the presence of other links of this kind in addition to the well-known auriculo-ventricular bundle. These investigations are of the greatest importance, and are likely to assist materially the cardiological work that is being done in the recently constituted heart laboratories in London and elsewhere.

Dr. F. Parkes Weber, M.D., F.R.C.P., has lately brought to the notice of the Royal Society of Medicine an interesting case of spasmodic stricture of the œsophagus which eventually had a fatal ending. Cases of stricture of the œsophagus in which no organic disease is present and in which the narrowing appears to be dependent on some muscular spasm, or faulty working of the nerve-muscle mechanism of swallowing, are by no means common, but it is extremely important that when they do occur those from whom they seek assistance should understand the nature of the condition so as to prevent death if possible. The best treatment appears to be the passage of a soft tube from time to time with the object of dilating the stricture, although as in Dr. Weber's case the very greatest care and expert knowledge may not remedy the malady. There are a number of specimens illustrating this kind of stricture in the pathological museum at Guy's Hospital.

A St. Thomas's man, Dr. A. J. Scott Pinchin, has been chosen to fill the vacancy for an out-patient physician at the Hampstead General Hospital. Dr. Pinchin qualified M.R.C.S., L.R.C.P. in 1906, and distinguished himself by winning a gold medal in medicine at the M.D. London examination five years ago. After qualification he was casualty officer, resident anæsthetist, and house physician at St. Thomas's; subsequently he joined the School Medical Staff of the L.C.C., to which he was appointed assistant school medical officer.

Dr. J. Parlane Kinloch, who has been elected to the new lectureship in public health at Aberdeen University, is an M.B., B.Ch. Glasg., and D.P.H. Cantab. Since qualifying he has held the appointments of house surgeon and house physician to the Throat, Nose, and Ear Victoria Infirmary at Glasgow, and extern resident medical officer at Queen Charlotte's Hospital. Latterly, having devoted himself to public health work, he has been resident assistant physician at the Ruchill Fever Hospital, Glasgow.

